# Development and Treatments of Inflammatory Cells and Cytokines in Spinal Cord Ischemia-Reperfusion Injury

**DOI:** 10.1155/2013/701970

**Published:** 2013-07-14

**Authors:** Ping Zhu, Jia-xin Li, Masayuki Fujino, Jian Zhuang, Xiao-Kang Li

**Affiliations:** ^1^Guangdong Cardiovascular Institute, Guangdong General Hospital, Guangdong Academy of Medical Sciences, 106 Zhongshan Er Road, Guangzhou 510100, China; ^2^National Research Institute for Child Health and Development, 2-10-1 Okura, Setagaya-ku, Tokyo 157-8535, Japan; ^3^National Institute of Infectious Diseases, Tokyo, Japan

## Abstract

During aortic surgery, interruption of spinal cord blood flow might cause spinal cord ischemia-reperfusion injury (IRI). The incidence of spinal cord IRI after aortic surgery is up to 28%, and patients with spinal cord IRI might suffer from postoperative paraplegia or paraparesis. Spinal cord IRI includes two phases. The immediate spinal cord injury is related to acute ischemia. And the delayed spinal cord injury involves both ischemic cellular death and reperfusion injury. Inflammation is a subsequent event of spinal cord ischemia and possibly a major contributor to spinal cord IRI. However, the development of inflammatory mediators is incompletely demonstrated. And treatments available for inflammation in spinal cord IRI are insufficient. Improved understanding about spinal cord IRI and the development of inflammatory cells and cytokines in this process will provide novel therapeutic strategies for spinal cord IRI. Inflammatory cytokines (e.g., TNF-**α** and IL-1) may play an important role in spinal cord IRI. For treatment of several intractable autoimmune diseases (e.g., rheumatoid arthritis), where inflammatory cytokines are involved in disease progression, anti-inflammatory cytokine antagonist is now available. Hence, there is great potential of anti-inflammatory cytokine antagonist for therapeutic use of spinal cord IRI. We here review the mediators and several possibilities of treatment in spinal cord IRI.

## 1. Inflammation in Spinal Cord IRI

Inflammation is a subsequent event of spinal cord ischemia and a plausible pathway in spinal cord ischemia-reperfusion injury (IRI) [1]. A series of metabolic processes ensue following ischemia. In a porcine model of 45-minute thoracoabdominal aortic occlusion, a strong immune response, which manifested as hyperemia and accumulation of inflammatory cells, occurred 48 h after the end of the aortic occlusion [2]. In a rabbit model of spinal cord ischemia, prominent inflammatory cell infiltration was observed [3]. These studies indicate that ischemia initiates an inflammatory reaction in the spinal cord.

Restoration of spinal cord blood flow would lead to so called reperfusion injury [4], which has been known as a biochemically mediated event [5]. Restored blood flow stimulates expression of adhesion molecules and chemokines, resulting in inflammatory reaction that involves neurotoxicity, recruitment of leucocytes, polymorphonuclear microvessel endothelial damage, hypoperfusion, and apoptosis [6]. In a swine model of spinal cord IRI, neutrophil sequestration and neuronal viability changed within 24 hours of reperfusion [7]. In a rat model of spinal cord IRI, researchers observed inflammatory cell infiltration in the gray matters of the spinal cords [1]. Delayed motor neuron death was detected during the same period as the strong immune response in the gray matter [2]. However, in a murine model of thoracic aortic ischemia reperfusion, there was no correlation between markers of inflammation and neurologic outcomes [8]. These observations indicated that inflammation might be a major contributor to spinal cord IRI, especially in the reperfusion period.

## 2. Inflammatory Cells in Spinal Cord IRI

Inflammatory response in spinal cord IRI was characterized by a massive accumulation of inflammatory cells in the gray matter [2]. Inflammatory cells in spinal cord IRI mainly include macrophages, lymphocytes, neutrophils, microglia, and astrocytes [9]. They were usually observed as perivascular infiltration cells in spinal cord IRI [9]. Kiyoshima T demonstrated that delayed onset paraplegia was largely associated with necrotic cell death with prominent inflammatory cell infiltration [10]. However, little is known about the activation and reaction of these inflammatory cells in spinal cord IRI.

### 2.1. Macrophages

In a rat model of spinal cord IRI, a number of bone marrow-derived macrophages were present 7d after IRI [11]. In the animals that suffered from severe paraplegia, a robust accumulation of bone marrow-derived macrophages occupied the entire ischemic gray matter [11]. In a rabbit model of spinal cord IRI, macrophages were first detected at 8 hours after reperfusion and mainly surrounded the infarction area [12].

### 2.2. Neutrophils

Activated neutrophils play a key role in the development of spinal cord IRI [13]. Accumulation of neutrophils in the postischemic spinal cord tissue could be evaluated by measuring myeloperoxidase (MPO) levels. In a rat model of spinal cord IRI, spinal cord tissue levels of MPO were increased after spinal cord IR, peaking at 24 h after reperfusion [14]. In a rat model of spinal cord IRI, tissue MPO activity (mean 0.60 ± 0.046 U/g) increased significantly at 24 h after reperfusion, compared with the control group (mean 0.23 ± 0.040 U/g) [9].

### 2.3. Microglia

Microglia are the resident immune cells of the central nervous system [15]. They could be activated early after spinal cord reperfusion injury and share many immunological characteristics with peripheral macrophage [12, 15]. Researches indicate that the proliferation and activation of microglia contributes to excitotoxicity [16], which is an important mechanism of spinal cord IRI. Olson examined the immune response by microglia in the spinal cord; their observations revealed that microglia in the spinal cord of mice expressed higher levels of surface immune molecules and may have different immune reactivity which may contribute to spinal cord diseases [15].

### 2.4. Astrocytes

Astrocytes are one of the major components of the blood-brain (spinal cord) barrier and play a role in the development of spinal cord IRI and its neurological outcomes. In a rabbit model of spinal cord IRI, astrocytes were activated early (2 hours) after reperfusion in the gray matter of the lumbar spinal cord, but confined to the area where neurons started to show degeneration [12]. This finding suggested that astrocytes might be important in the mechanism of delayed onset motor dysfunction in spinal cord IRI.

## 3. Cytokines in Spinal Cord IRI

Spinal cord IRI is correlated to increases in inflammatory chemokines release [17]. Several cell types have been shown to synthesize inflammatory cytokines [1]. Experiments in animal models of spinal cord IRI revealed that macrophages and microglia strongly expressed tumor necrosis factor-*α* (TNF-*α*), interleukin 1*β* (IL-1*β*), and other mediators [7, 11, 16]. In a rat model of spinal cord IRI, strong staining of IL-1*β*, IL-10, and TNF-*α* was observed, suggesting a dramatic infiltration of inflammatory cells in both gray matters and peripheral white matters [18].

Cytokines are a group of proteins produced during the activation of inflammatory response [1] and play an important role in the subsequent spinal cord IRI. However, data about the pathways and effects of these cytokines in the spinal cord IRI are still limited [9]. In rats that suffered from severe paraplegia induced by spinal cord IRI, TNF-*α*, IL-1*β*, and other mediators were strongly expressed [11]. Lu and colleagues discovered that the mitogen-activated protein kinase/extracellular signal-regulated kinase (MEK/ERK) pathway might play a noxious role in spinal cord IRI via participating in inflammatory reactions and cytokine production [19].

Species and alternation of cytokines in spinal cord IRI have not been well determined. Smith and colleagues analyzed 23 cytokines in a mice model of spinal cord IRI, and the chemokines IL-1, IL-6, keratinocyte chemoattractant (KC; murine equivalent of human IL-8), and TNF-*α* increased significantly and showed a biphasic response [17]. However, Kunihara and colleagues discovered that levels of TNF-*α*, IL-1*β*, IL-6, and IL-12 in serum and cerebrospinal fluid (CSF) did not significantly change [20].

### 3.1. TNF-*α*

TNF-*α* is a potent activator of neutrophils [21]. TNF-*α* binds to two receptors: type 1 TNF receptor (p55) and type 2 TNF receptor (p75), which are expressed in many types of cells [22].

TNF-*α* increased significantly in reperfusion period and remained at high level after reperfusion. In a rabbit model of 30 minutes aortic occlusion and 2 hours of reperfusion, TNF-*α* level was significantly increased to 120.44 ± 8.95 pg/mg protein in the IRI group compared with the sham group (25.34 ± 1.03 pg/mg protein) [4].

Besides significant elevated expression, TNF-*α* showed trends toward a biphasic, early and late, peak in expression [17]. In a rat model of spinal cord IRI (a balloon catheter placed into the aorta), TNF-*α* level was significantly increased within 1.5 hours after the transient ischemia and peaked at 3 hours [21]. In a swine model of spinal cord IRI, TNF-*α* levels increased significantly by 6 hours to 12 hours after reperfusion, suggesting a similar pattern of bimodal release of TNF-*α* after IRI [7].

The high levels of TNF-*α* persisted for a long time after second expression peak. In a rat model of spinal cord IRI, TNF-*α* levels in the 24 hours sham operated group and 24 hours IRI group were 48.30 ± 11.11 pg/mL and 138.62 ± 78.58 pg/mL, respectively [1]. TNF-*α* levels in the 48 hours sham operated group and 48 hours IRI were 68.65 ± 25.15 pg/mL and 129.16 ± 51.27 pg/mL, respectively. There were no significant changes for TNF-*α* levels between the 24 hours and 48 hours after IRI (*P* > 0.05) [1]. In a rat model of spinal cord IRI, spinal cord sections from the 48 hours reperfusion group exhibited a strong positive staining for TNF-*α*, mainly localized in various cells in the gray matter [23]. In a rat model of spinal cord IRI, the serum levels of TNF-*α* increased from 257 to 629 pg/mL at 24 hours after IRI [24]. In a rabbit model of spinal cor IRI, TNF-*α* expression at 1.5 hours (53.4 ± 12.3) and 3 days (92.4 ± 5.7) of reperfusion was higher than at 5 days (40.4 ± 20.1), and at 3 days it was higher than at 1.5 h [25].

Excitotoxic cell death due to glutamate release is important in the secondary injury following spinal cord ischemia [26]. Neurophysiological studies show that TNF-*α* can potentiate the effects of glutamatergic afferent input to produce hyperactivation of neurons [26]. These results suggest that proinflammatory cytokines, especially TNF-*α* might contribute to excitatory cell death in spinal cord IRI.

### 3.2. Interleukin 1 (IL-1)

IL-1 family cytokines include the secreted proinflammatory agonist IL-1*β*, IL-18, and anti-inflammatory receptor antagonist IL-1a [9]. IL-1*β* has been implicated in extensive inflammation and progressive neurodegeneration after ischemia [10]. In a rat model of spinal cord IRI, spinal cord sections from the 48 hours reperfusion group exhibited a strong positive staining for IL-1, mainly localized in various cells in the gray matter [23].

IL-1 also showed a biphasic expression in spinal cord IRI. In a mice model of spinal cord IRI, IL-1 expression was significantly increased at 6 hours and 36 hours into reperfusion [17]. IL-1 expression at 6 hours and 36 hours was also increased compared with 18 hours, signifying a biphasic response to reperfusion [17].

IL-1*β* levels increased significantly in reperfusion period and remained at high levels after reperfusion. In a rat model of spinal cord IRI, IL-1*β* levels in the 24 hours sham operated group and 24 hours IRI group were 22.21 ± 8.64 pg/mL and 58.01 ± 26.46 pg/mL, respectively [1]. IL-1*β* levels in the 48 hours sham operated group and 48 hours IRI group were 31.45 ± 16.43 pg/mL and 71.65 ± 15.90 pg/mL, respectively. There were no significant changes for IL-1*β* levels between 24 hours IRI group and 48 hours IRI group (*P* > 0.05) [1]. In a rat model of spinal cord IRI, the mean spinal cord IL-1 amounts were 20.38 ± 2.49 at 1 day and 19.69 ± 3.21 at 3 days [19].

MEK/ERK pathway might play a noxious role in spinal cord IRI via participating in inflammatory reactions and cytokine production [19]. In a rat model of spinal cord IRI, MEK/ERK pathway inhibition with U0126, highly selective inhibitor of both MEK1 and MEK2 (MEK1/2) [27], dramatically reduced microglia accumulation and IL-1 expression, resulting in improved neuronal survival [19]. This study suggested a role of the MEK/ERK pathway in the inflammatory responses after spinal cord IRI might be partly mediated by its inhibitory effects on microglia activation and IL-1 production [19].

### 3.3. Interleukin 6 (IL-6)

IL-6 is a proinflammatory cytokine induced by spinal cord IRI and increased significantly in the process of IRI. In a mice model of spinal cord IRI, IL-6 was significantly increased compared with all other time points and peaked at 36 hours, without significant increase in expression at other time points [17]. In a rabbit model of aortic occlusion and reperfusion, IL-6 levels were significantly increased to 87.40 + 5.86 pg/mg protein after IRI, compared with 11.46 + 1.09 pg/mg protein in the sham group [4].

The increase in IL-6 expression continued for hours after reperfusion. In a rat model of spinal cord IRI, IL-6 levels in the 24 hours sham operated group and 24 hours IRI group were 48.21 ± 19.79 pg/mL and 372.50 ± 134.62 pg/mL, respectively. IL-6 levels in the 48 hours sham operated group and 48 hours IRI group were 216.51 ± 74.48 pg/mL and 847.20 ± 350.28 pg/mL, respectively. These data revealed that IL-6 levels at 48 hours after IRI were significantly higher than those at 24 hours (*P* < 0.05) [1].

### 3.4. Interleukin 8 (IL-8)

IL-8 levels might be correlated with neurological outcomes after spinal cord IRI. Kunihara and colleagues measured levels of cytokines in perioperative serum and CSF in fifteen adult patients undergoing aortic repair. IL-8 levels in CSF peaked after operation and maintained the higher levels for 72 hours. The patients with paraplegia had the highest IL-8 levels in CSF throughout the study period [20].

The increase in IL-8 levels showed a biphasic pattern in spinal cord IRI. In a mice model of spinal cord IRI, KC (murine equivalent of human IL-8) expression peaked at 6 hours and 36 hours, though the first peak was not marked enough to meet statistical significance [17]. KC expression peaked at 36 hours, meeting statistical significance when compared with that at 18 hours of reperfusion [17]. In a rat model of spinal cord IRI (a balloon catheter placed into the aorta), IL-8 level was increased and peaked at 12 hours after the transient ischemia. The biphasic expression of inflammatory cytokines would support a bimodal mechanism of spinal cord IRI.

### 3.5. Interleukin 10 (IL-10)

IL-10 is a potent anti-inflammatory cytokine induced by spinal cord IRI. In a rat model of spinal cord IRI, spinal cord sections from the 48 hours reperfusion group exhibited a strong positive staining for IL-10, mainly localized in various cells in the gray matter [23]. In a model of excitotoxic spinal cord injury induced by quisqualic acid, excitotoxic injury plus IL-10 treatment resulted in a significant downregulation of IL-1*β* and iNOS mRNA, suggesting that IL-10 could reduce spinal cord inflammation [28]. In a rat model of spinal cord IRI, MPO activity was slightly increased in IL-10-treated group (0.34 ± 0.029 U/g) with respect to control animals, suggesting that the administration of IL-10 could decrease IRI-induced MPO activity early after spinal cord IRI [9].

### 3.6. mRNAs

Inflammatory mRNAs are involved in the mediation of spinal cord IRI. In a rat model of spinal cord IRI [29], some anti-inflammatory mRNAs such as annexin A7 mRNA were potential targets of miR-323. Conversely, some inflammatory mediator mRNAs such as integrin, TNF-*α*, IL-1*β*, TNF receptor-associated factor 6, interleukin-1 receptor-associated kinase 1, and CD80 mRNAs were potential targets of miR-210, miR-146a, and miR-199a-3p, which were downregulated after spinal cord IRI [29].

## 4. Treatments for Inflammation in Spinal Cord IRI

There are several strategies applied for treatments of inflammation in spinal cord IRI, targeting at inflammatory cells, cytokines, and their receptors and pathways. However, most of these therapeutic strategies are insufficiently elucidated and needed further measurements.

### 4.1. Adenosine A_**2A**_ Receptor Activation

Adenosine A_2A_ receptor activation might attenuate spinal cord inflammation [7]. In a swine model of spinal cord IRI, adenosine A_2A_ receptor activation attenuates every aspect of IRI [7]. In a mice model of aortic aneurysm formation following elastase perfusion, data suggest that A_2A_R is functionally active in mediating immune cell recruitment and protease expression in the medial and adventitial layers of the aortic wall during aortic aneurysm formation [30].

Systemic ATL-146e, a selective adenosine A_2A_ agonist, has been shown to reduce paralysis after spinal cord ischemia [31]. In a rabbit model of spinal cord IRI (45-minute cross-clamping of the infrarenal aorta), ATL-146e reduced spinal cord reperfusion injury probably by reducing circulating TNF-*α* during a critical 3 h reperfusion interval [31].

### 4.2. Pentoxifylline

Pentoxifylline is an inhibitor of TNF-*α*. Since TNF-*α* is an important contributor to spinal cord IRI which might induce necrosis and apoptosis of cells, its inhibitor might exhibit a protective role in spinal cord injury following ischemia [32]. In a rabbit model of spinal cord IRI (45 minutes cross-clamping of the infrarenal aorta), a significant decrease in both necrotic and apoptotic neurons was observed in the Pentoxifylline-treated groups compared with the IRI group (*P* < 0.05) [32].

### 4.3. U0126

U0126 is a specific inhibitor of MAPK/ERK kinases 1/2 (MEK1/2) [27]. In a rat model of spinal cord IRI, the IL-1 levels in the U0126 group were significantly lower than those in the control group (*P* = 0.021), suggesting that MEK/ERK inhibition with U0126 might reduce microglia accumulation and IL-1 expression [19]. A further study indicated that pretreatment with U0126 inhibited ERK1/2 phosphorylation and significantly attenuated apoptosis and increased neuronal survival [27].

### 4.4. Infliximab

Infliximab is a humanized mouse monoclonal antibody to TNF-*α* [22]. In a rabbit model of spinal cord IRI, the infliximab group had significantly less vascular proliferation, edema, and neuron loss than the I/R group.

### 4.5. IL-1ra

IL-1ra is receptor antagonist and has anti-inflammatory properties and was expected to suppress inflammatory response in spinal cord IRI [10]. In a rabbit model of spinal cord IRI (aortic cross-clamping), a higher number of viable neurons were observed with less severe spinal cord injury in IL-1ra group (*P* < 0.01 at 24 hrs, *P* < 0.05 at 72 hrs, and *P* < 0.05 at 120 hrs). TUNEL-positive neurons were also significantly reduced by the administration of IL-1ra (*P* < 0.01 at 24 hrs and *P* < 0.05 at 120 hrs) [10]. These studies indicated that IL-1-targeted anticytokine therapy could be a potential strategy for improving the neurological outcomes after spinal cord IRI [10].

### 4.6. Activated Protein C (APC)

It is reported that APC might reduce spinal cord injury in rats by inhibiting neutrophil activation after the transient ischemia [21]. In a rat model of spinal cord IRI (a balloon catheter placed into the aorta), the increases in the tissue levels of TNF-*α*, rat IL-8, and myeloperoxidase in the ischemic part of the spinal cord were significantly reduced in animals that received APC [21].

### 4.7. Statins

Statins are the 3-hydroxy-3-methylglutaryl coenzyme A reductase inhibitors and might have pleiotropic effects that are independent of cholesterol lowering, such as anti-inflammatory effects, antioxidant effects, and endothelial function improvement [29].

### 4.8. Tetramethylpyrazine (TMP)

TMP is a pure compound derived from Ligusticum chuanxiong, which is widely applied for the treatment of ischemic stroke. Recent studies indicated that TMP might also exert neuroprotective effects on inflammation in spinal cord IRI. In a rat model of spinal cord IRI [23], treatment with TMP considerably reduced the degree of positive IL-1 and TNF-*α* staining and increased the degree of positive IL-10 staining in the spinal cord. Compared with the findings from the sham group, the expression levels of TNF-*α*, IL-1, and IL-10 in the spinal cord were significantly increased in the control group. The increase in TNF-*α* and IL-1 was significantly attenuated by TMP treatment (*P* < 0.01). Correspondingly, there was a significant elevation in the expression level of anti-inflammatory IL-10 in the TMP group (*P* < 0.01) [23].

### 4.9. Hydrogen

Hydrogen gas is a new popular therapeutic agent for tissue IRI. In a rabbit model of spinal cord IRI, the beneficial effects of hydrogen gas treatment against spinal cord IRI were associated with the decreased levels of proinflammatory cytokines (TNF-*α*) in serum and spinal cord [33].

### 4.10. Glycyrrhizin

Glycyrrhizin is a natural triterpene glycoconjugate derived from the root of licorice (Glycyrrhiza glabra). Glycyrrhizin might attenuate the transient spinal cord ischemic injury in rats via reducing inflammatory cytokines. In a rat model of spinal cord IRI, Glycyrrhizin reduced levels of TNF-*α*, IL-1*β*, and IL-6 in the plasma and injured spinal cord [34].

### 4.11. Panax Notoginsenoside

Panax notoginsenoside is an important traditional Chinese medicine and might exert potent neuroprotective effects on spinal cord IRI probably by its antiinflammation, antiedema, and antiapoptosis actions. In a rat model of spinal cord IRI, after Panax notoginsenoside or Methylprednisolone treatment, we observed reduced immunostaining of IL-1*β*, IL-10, and TNF-*α*, indicating that the infiltration of inflammatory cells was greatly relieved [18].

### 4.12. Intrathecal Transplantation of Bone Marrow Stromal Cells

In a rabbit model of spinal cord IRI, transplantation of bone marrow stromal cells reduced the excessive expression of TNF-*α* (*P* < 0.01) [35].

### 4.13. Ghrelin

In a rat model of spinal cord IRI, administration of ghrelin significantly attenuated the serum TNF-*α* level [24].

### 4.14. Thalidomide

Intraperitoneal (i.p.) administration of thalidomide might reduce IRI in a rabbit spinal cord model via reduction of TNF-*α* expression [25].

### 4.15. Antithrombin (AT)

Antithrombin (AT) significantly inhibited the IR-induced increases in spinal cord tissue levels of TNF-*α*, rat IL-8, and myeloperoxidase [36].

### 4.16. Diltiazem

Diltiazem has cytoprotective and anti-inflammatory properties, leading to reduced spinal cord injury. In a rabbit model of spinal cord IRI (30-minute infrarenal aortic occlusion), diltiazem infusion significantly reduced IL-6 levels at 3 h and 24 h after reperfusion, and the mean IL-10 level in the diltiazem group was significantly higher than in the control group at 24 h after reperfusion [37].

### 4.17. Lazaroids

Lazaroids is 21-aminosteroids that might affect the production of both proinflammatory and anti-inflammatory cytokines in spinal cord IRI. In a rabbit model of spinal cord IRI (20-minute infrarenal aortic cross-clamping), plasma IL-8 and IL-1ra levels in lazaroids group were significantly lower than other groups (*P* < 0.05). Spinal IL-8 levels in lazaroids group (0.98 ± 0.34 ng/g tissue) were lower than those in control group (7.26 ± 2.26 ng/g tissue) (*P* < 0.05) [38].

### 4.18. Potential of Clinical Use

As reviewed in Section 3 “Cytokines in Spinal Cord IRI,” inflammatory cytokines play an important role in spinal cord IRI as mediator. For treatment of several intractable autoimmune diseases (e.g., rheumatoid arthritis), where inflammatory cytokines are involved in disease progression, anti-inflammatory cytokine antagonist is now available. Hence, there is great potential of anti-inflammatory cytokine antagonist for therapeutic use of spinal cord IRI. Infliximab and other anti-TNF-*α* monoclonal antibodies are used mainly for treatment for autoimmune diseases [39]. The acute infusion reactions are well known as adverse effect of the monoclonal antibody therapy [40]. Therefore, the managing premedications for reactions to infusional monoclonal antibody therapy are mandatory [41, 42]. Among other inhibitors of inflammatory cytokines, IL-1ra is used to treat the symptoms of moderate to severe rheumatoid arthritis [43]. The most common side effect has included injection site reactions [44]. Besides the monoclonal antibodies of anti-inflammatory cytokines, Pentoxifylline, other TNF-*α* inhibitors, is already used clinically for treatment of intermittent claudication in certain patients to reduce pain, cramping, numbness, or weakness in the arms or legs and has been generally well tolerated. There are typically minor side effects for approximately 3% of treated patients with continuation of the drug.

Amongst more broad anti-inflammatory agents, ATL-146e is now in Phase III clinical trial as a pharmacological stress agent for use in myocardial perfusion imaging. ATL146e is more selective for A_2A_ receptor than CGS21680 and therapeutically more interesting, with lower side effects [45].

Instead of pharmaceutical drugs, the medical gases, nitric oxide, carbon monoxide, and hydrogen sulfide, have traditionally been considered to be toxic and environmentally hazardous, being now considerable therapy for spinal cord IRI. The numerous experimental animal and human studies of these agents have demonstrated protective effects against IRI. Effects of hydrogen have been reported in more than 60 disease models and human disease [46]. Only two diseases of cerebral infarction and metabolic syndrome have been analyzed in both animals and humans. It is noteworthy that, however, lack of any adverse effects of hydrogen enabled clinical studies even in the absence of animal studies.

## 5. Ineffective Treatments for Inflammation in Spinal Cord IRI

As researchers have attempted to discover more treatments for inflammation in spinal cord IRI, there are some strategies which were proved to be ineffective.

### 5.1. Heparin

The recently discovered anti-inflammatory property of glycosaminoglycans, including heparin, deserves to be investigated. However, in a rat model of spinal cord IRI, there was no significant difference between the groups in terms of the degree of inflammatory response, degree of IL-6, HSP-70, or MPO staining [47].

### 5.2. Carbamylated Erythropoietin Fusion Proteins or Recombinant Human Erythropoietin

In a swine model of spinal cord IRI, neither carbamylated erythropoietin fusion protein nor recombinant human erythropoietin affected the rise in IL-6 and TNF-*α* levels, and infiltration of inflammatory cells into the spinal cord did not show any intergroup difference [48].

## 6. Summary and Conclusion

Spinal cord IRI includes acute ischemic injury and delayed reperfusion injury. Inflammation is a subsequent event in both periods and a major contributor to spinal cord IRI. Inflammatory cells in spinal cord IRI mainly include macrophages, lymphocytes, neutrophils, microglia, and astrocytes. Inflammatory cells might participate in spinal cord IRI by inducing cell death and expressing inflammatory cytokines. Cytokines involved in spinal cord IRI include TNF-*α*, IL-1, IL-6, IL-8, and IL-10. Apart from IL-10, cytokines are characterized as proinflammation factors. The biphasic elevated expression of TNF-*α*, IL-1, and IL-8 might suggest a bimodal mechanism of spinal cord IRI. The effects of MEK/ERK pathway on inflammation might be mediated by inhibiting microglia activation and IL-1 production. Development and reactions of these inflammatory mediators should be fully elucidated. Diverse therapeutic strategies have been discovered for reducing inflammatory cells and cytokines in spinal cord IRI, including Adenosine A_2A_ receptor activation, inhibitor and antibody of TNF-*α*, IL-1 receptor antagonist and pathway inhibition, and other agents. The mechanisms and potency of these strategies deserve to be further demonstrated, in order to provide safer and more effective treatments applied for clinical practice.
